# Supplementary material for: Pollen report: quantitative review of pollen crude protein concentrations offered by bee pollinated flowers in agricultural and non-agricultural landscapes
Source: PeerJ. 2019 Aug 12;7:e7394. doi: 10.7717/peerj.7394 (PMC6694784; doi:10.7717/peerj.7394)
Supplement: Table S1 — Comparision of genus level pollen quality patterns based on all observations and species/variety mean values. [file peerj-07-7394-s001.docx]

| Table S1: Summarizes the total number observations and species (wild & weed) or varieties (crop) per genus. In addition we present the mean protein concentration based on all observations or species/variety means. | | | | |
| --- | --- | --- | --- | --- |
| Genus | Observations | Mean protein concentration (based on all observations) | Species/Varieties | Mean protein concentration (based on species/variety mean) |
|  |  |  |  |  |
| Acacia | 4 | 23.75 | 3 | 23.73 |
| Acer | 3 | 32.53 | 3 | 32.53 |
| Arctotheca | 3 | 18.37 | 1 | 18.40 |
| Banksia | 4 | 31.25 | 2 | 31.30 |
| Brassica | 12 | 28.10 | 12 | 28.10 |
| Castanea | 3 | 22.87 | 3 | 22.87 |
| Casuarina | 3 | 13.43 | 1 | 13.40 |
| Centaurea | 5 | 23.46 | 2 | 23.75 |
| Cirsium | 4 | 23.85 | 2 | 25.35 |
| Cistus | 3 | 14.97 | 3 | 14.97 |
| Cornus | 3 | 21.13 | 3 | 21.13 |
| Corymbia | 4 | 29.13 | 2 | 28.40 |
| Cylindropuntia | 6 | 26.35 | 6 | 26.35 |
| Echium | 5 | 33.32 | 2 | 33.90 |
| Eucalyptus | 35 | 24.78 | 19 | 25.33 |
| Helianthus | 8 | 17.60 | 8 | 17.60 |
| Hypochoeris | 4 | 16.55 | 1 | 16.60 |
| Lupinus | 3 | 31.60 | 3 | 31.60 |
| Papaver | 3 | 25.07 | 3 | 25.07 |
| Prunus | 7 | 31.39 | 6 | 31.72 |
| Rapistrum | 3 | 24.90 | 1 | 24.90 |
| Rubus | 4 | 23.78 | 4 | 23.78 |
| Salix | 5 | 27.48 | 4 | 28.10 |
| Senna | 5 | 44.62 | 5 | 44.62 |
| Sinapis | 5 | 26.42 | 4 | 25.86 |
| Solanum | 19 | 46.63 | 19 | 46.63 |
| Trifolium | 13 | 27.32 | 13 | 27.32 |
| Vicia | 5 | 29.42 | 3 | 31.27 |
| Zea | 8 | 17.78 | 8 | 17.78 |
|  |  |  |  |  |
